# Extracellular Matrix Collagen I Differentially Regulates the Metabolic Plasticity of Pancreatic Ductal Adenocarcinoma Parenchymal Cell and Cancer Stem Cell

**DOI:** 10.3390/cancers15153868

**Published:** 2023-07-29

**Authors:** Diana Tavares-Valente, Stefania Cannone, Maria Raffaella Greco, Tiago Miguel Amaral Carvalho, Fátima Baltazar, Odília Queirós, Gennaro Agrimi, Stephan J. Reshkin, Rosa Angela Cardone

**Affiliations:** 1 Life and Health Sciences Research Institute (ICVS), School of Medicine, University of Minho, 4710-057 Braga, Portugal; dvalente@ucp.pt; 2 ICVS/3B’s—PT Government Associate Laboratory, 4805-017 Braga, Portugal; 3 UNIPRO—Oral Pathology and Rehabilitation Research Unit, University Institute of Health Sciences, CESPU, CRL, 4585-116 Gandra, Portugal; odilia.queiros@iucs.cespu.pt; 4 Department of Biosciences, Biotechnology and Environment, University of Bari, 70125 Bari, Italy; stefaniacannone92@gmail.com (S.C.); grecoraffaella1975@gmail.com (M.R.G.); tiagomac94@gmail.com (T.M.A.C.); gennaro.agrimi@uniba.it (G.A.); rosaangela.cardone@uniba.it (R.A.C.)

**Keywords:** pancreatic ductal adenocarcinoma, tumor microenvironment, chemoresistance, treatment, cancer stem cells, collagen I, bioenergetic modulators, glutamine

## Abstract

**Simple Summary:**

Pancreatic ductal adenocarcinoma (PDAC) has an extremely poor prognosis largely due to the intense fibrotic desmoplastic reaction, characterized by high levels of extracellular matrix (ECM) collagen I that constitutes a niche for the cancer stem cells (CSCs). The role of the ECM composition in determining metabolic plasticity is still unknown. As ECM collagen I content increased, the CSCs switched from glucose to mostly glutamine metabolism. While all the bioenergetic modulators (BMs) decreased cell viability and increased cell death in all extracellular matrix types, a distinct, collagen I-dependent profile was observed in CSCs, in which the CSCs switched from glucose to mostly glutamine metabolism. Furthermore, all BMs synergistically potentiated the cytotoxicity of paclitaxel albumin nanoparticles (NAB-PTX) in both cell lines.

**Abstract:**

Pancreatic ductal adenocarcinoma (PDAC) has a 5-year survival rate of less than 10 percent largely due to the intense fibrotic desmoplastic reaction, characterized by high levels of extracellular matrix (ECM) collagen I that constitutes a niche for a subset of cancer cells, the cancer stem cells (CSCs). Cancer cells undergo a complex metabolic adaptation characterized by changes in metabolic pathways and biosynthetic processes. The use of the 3D organotypic model in this study allowed us to manipulate the ECM constituents and mimic the progression of PDAC from an early tumor to an ever more advanced tumor stage. To understand the role of desmoplasia on the metabolism of PDAC parenchymal (CPC) and CSC populations, we studied their basic metabolic parameters in organotypic cultures of increasing collagen content to mimic in vivo conditions. We further measured the ability of the bioenergetic modulators (BMs), 2-deoxyglucose, dichloroacetate and phenformin, to modify their metabolic dependence and the therapeutic activity of paclitaxel albumin nanoparticles (NAB-PTX). While all the BMs decreased cell viability and increased cell death in all ECM types, a distinct, collagen I-dependent profile was observed in CSCs. As ECM collagen I content increased (e.g., more aggressive conditions), the CSCs switched from glucose to mostly glutamine metabolism. All three BMs synergistically potentiated the cytotoxicity of NAB-PTX in both cell lines, which, in CSCs, was collagen I-dependent and the strongest when treated with phenformin + NAB-PTX. Metabolic disruption in PDAC can be useful both as monotherapy or combined with conventional drugs to more efficiently block tumor growth.

## 1. Introduction

Pancreatic ductal adenocarcinoma (PDAC) is the fourth cause of death from cancer in Western countries [1]. In most cases, it is diagnosed at an advanced stage and only a low percentage of patients are able to undergo surgical resection. PDAC presents a 5-year survival rate of less than 10%, due to the development of early metastases [2]. PDAC is characterized by an extensive desmoplasia of the extracellular matrix (ECM), characterized by the accumulation of collagen I [3,4,5,6,7,8]. The fibrotic and dense stroma is responsible for poor tumor vascularization, leading to hypoxic regions [9,10] associated with metastatic potential, as well as resistance to chemo- and radiotherapy [6,8,11,12]. The first-line chemotherapeutic drug is gemcitabine, combined either with paclitaxel associated with albumin (NAB-PTX) or with FOLFIRINOX (a combination of 5-fluorouracil, leucovorin, oxaliplatin and irinotecan) [13,14]. However, both regimens have low efficacy rates [15]. Another feature responsible for treatment failure and tumor relapse is the presence of a subset of cells with stem cell characteristics [16,17], the cancer stem cells (CSCs). CSCs have been identified in different types of cancers, such as leukemia [18], glioma [19], colon [20], lung [21], breast [22], prostate cancers [23] and also in PDAC [24,25,26]. They present similar features to normal stem cells, such as self-renewal capacity and the competence to differentiate into multiple cancer cell types, a functional system of DNA repair, metabolic adaptations and other stemness abilities [27]. Thus, the development of new therapies that preferentially target CSCs could result in important improvements in current antitumor strategies in PDAC patients [26]. 

One of the features that can be used as a promising target is the reprogrammed metabolism [28]. The non-stem parenchymal pancreatic cancer cells (CPCs) show an increased dependence on glycolysis coupled with a high production of lactate, known as the Warburg effect [29]. This metabolic switch induces the upregulation of glycolytic enzymes, glucose transporters (GLUTs) and lactate transporters (MCTs) and there are reports of the overexpression of these transporters in PDAC [30,31]. While non-tumoral stem cells rely more on glycolysis than differentiated cells that prefer OXPHOS [32], there are a few studies published about the metabolic features of CSCs with conflicting/controversial results and explanations. Even if glucose is considered to be the main metabolite used by CSCs, they can use it to fuel different metabolic pathways [16] and there are studies showing CSCs to be particularly reliant on amino acid metabolism for oxidative phosphorylation, especially in leukemia stem cells, which are completely dependent on metabolizing amino acids for energy [33,34]. While PDAC is characterized by high levels of glutamine uptake [35,36,37,38,39], neither the tumor cell type involved nor the role of the desmoplastic reaction in driving this glutamine uptake have been described. Many studies have revealed that CSCs identified in some types of cancer, such as PDAC [40], ovarian [41] and gliomas [42], rely mainly on oxidative metabolism with a higher mitochondrial mass content and higher oxygen consumption levels [43,44,45,46].

In contrast, other studies reported a more glycolytic profile, when these cells are maintained in hypoxic regions, regulated by HIF-1α and HIF-2α and, consequently, by an acid tumor microenvironment, responsible for stemness features and resistance to treatment [47]. Some examples are osteosarcoma [48], breast, lung [49] and colon cancers [50]. 

An explanation for different theories on the metabolic features of CSCs is likely to be due to their metabolic plasticity [51] that permits CSCs to adapt to the availability of nutrients and to external tumor environmental conditions [52]. This adaptive metabolic plasticity might allow CSCs to survive in variable, hostile environments found during tumor progression and there is no doubt that the altered metabolic profiles represent an emergent hallmark of CSCs that can be used to develop new therapeutic approaches. This would be an efficient strategy to eradicate the tumor cell population most responsible for tumor relapse and chemoresistance, thus helping to improve the survival rate of cancer patients [28]. Some studies have shown that CSCs are sensitive to OXPHOS inhibition by biguanide compounds, such as metformin, that inhibits ATP formation via the blockage of complex I in mitochondria [53,54]. Indeed, it has been shown that metformin enhanced the capacity of gemcitabine to inhibit the proliferation and invasion of PDAC cells by inhibiting the proliferation of CSC cell populations [55]. However, recent studies using metformin produced somewhat disappointing results [56,57], which has led to a new clinical trial using another more potent/efficacious biguanide compound, phenformin, that showed promising results, but not in PDAC [58]. Glycolysis inhibitors, such as 2-deoxyglucose or 3-bromopyruvate, have also been tested in in vitro models of PDAC and both were able to decrease cell viability and increase cell death [45,59,60,61]. The limited success of the existent therapeutic modalities in pancreatic cancer has underlined the potential future importance of using metabolic inhibitors either singly or in combination with existing therapeutic regimens [45].

Unfortunately, the knowledge in this field is scarce and more efforts are needed to understand the benefit of using the altered metabolism as targets in PDAC treatment, including as co-adjuvant therapy. However, the existing studies of in vitro anti-metabolic treatments used 2D and not three-dimensional (3D) culture systems and did not take into account the influence of the extracellular desmoplastic environment predominant in PDAC [62]. Therefore, the efficacy of therapeutic strategies was not correctly evaluated. In this line, recent studies have demonstrated the importance of the extracellular matrix (ECM) in regulating the behavior of tumor parenchymal and CSCs [63,64,65]. Indeed, recent studies in PDAC cells have demonstrated that the stromal ECM composition “per se” provides important cues that guide the expression of growth kinetics, morphology, invasion, chemosensitivity and the secretome profiles in both PDAC parenchymal cells (CPCs) [66,67,68] and CSCs [67,68]. The use of the 3D organotypic model in this study allowed us to manipulate the ECM constituents and mimic the progression of PDAC from an early tumor to an ever more advanced tumor stage [62]. We therefore used this 3D organotypic culture platform to determine the role of ECM composition on metabolic plasticity in PDAC CPCs and CSCs and their response to various metabolic pathway inhibitors (energy disruptor molecules or bioenergetic modulators; called here, BMs) [45] in the absence and presence of nab-paclitaxel (NAB-PTX) [46]. 

## 2. Materials and Methods

### 2.1. Cell Lines and Culture

Two PDAC cell lines were utilized, the parenchymal, parental PANC-1 cell line (hereon called CPC) and the CSC cell line derived from the parenchymal line. The PANC-1 line was obtained from the American Type Culture Collection. The CSC line was selected as described [67,68] using a selective medium, DMEM-F12 (US Biological Sciences, Swampscott, MA, USA) without serum but containing (per L) 10 mL B27 (Gibco, Life Technologies, New York, NY, USA), 1 ug/mL fungizone (Gibco, Life Technologies), 5 ug/mL heparin (Sigma Aldrich, St. Louis, MO, USA) and the growth factors EGF and FGF (20 ng/mL, PreProTech, Rocky Hill, CT, USA). After selection, the CSCs were maintained in this medium for all experiments. The CPC cell line (PANC-1) was maintained in RMPI (Gibco, Life Technologies) supplemented with 10% fetal bovine serum (FBS, Gibco, Life Technologies), 50 µg/mL gentamycin (Gibco, Life Technologies) and 1% penicillin–streptomycin solution (Gibco, Life Technologies). Both cell lines were maintained at 37 °C and 5% CO_2_.

### 2.2. Drugs and Their Application

The bioenergetic modulators (BMs) 2-DG (5 mM), DCA (20 mM) and phenformin (0.01 mM) (Sigma Aldrich) were dissolved in PBS at stock solutions of 1 M, 10 M and 100 mM, respectively. NAB-PTX was obtained from the University Hospital, Bari, Italy [69,70] at the final stock solution concentration of 5.8 mM, from which working solutions were prepared. Cells were plated into 96-well substrate-coated plates at a density of 1.5 × 10^3^ cells/well; after substrate polymerization and 24 h after cell plating, cells were treated with the chemotherapeutic drug, NAB-PTX, BMs (2-DG: 5 mM, DCA: 20 mM and phenformin: 0.01 mM) or a combination of both (each BM + NAB-PTX 10 nM).

### 2.3. Three Dimensional Organotypic Growth

Three different mixtures constituted by Matrigel Basement Membrane Matrix™ (Corning) and collagen I (bovine—Gibco, Life Technologies) were prepared as per [67,68]. The stock concentration of Matrigel was 7 mg/mL dissolved in serum-free media, whereas the stock concentration of collagen I was 3 mg/mL dissolved in distilled water, PBS 10X (Sigma Aldrich) and 0.015 N NaOH. Then, the mixtures were prepared as per [67,68]: 

A total of 90% Matrigel–10% collagen I. The mixtures of Matrigel and collagen I were prepared at the final concentration of 6.3 mg/mL and 0.3 mg/mL, respectively, as described above, and then mixed. 

A total of 70% Matrigel–30% collagen I. The mixtures of Matrigel and collagen I were prepared at the final concentration of 4.9 mg/mL and 0.9 mg/mL, respectively, as described above, and then mixed. 

A total of 10% Matrigel–90% collagen I. The mixtures of Matrigel and collagen I were prepared at the final concentration of 0.7 mg/mL and 2.7 mg/mL, respectively, as described above, and then mixed.

In 96-well plates, 100 µL of each ECM mixture was added to each well and, after 1 h of polymerization at 37 °C, all the wells were washed with a culture medium without serum, to eliminate the metabolites generated during the polymerization process. 

### 2.4. Cell Viability Assay

Cells were plated into 96-well substrate-coated plates at a density of 1.5 × 10^3^ cells/well, treated with the chemotherapeutic drugs as already described in Section 2.2 and cell viability was analyzed after 7 days in culture. The effect of compounds on cell proliferation was determined by the resazurin assay (alamarBlue^R^) (OD 590 nm), as described previously [62,67,68]. IC_50_ values were estimated from 3 independent experiments, each one in triplicate, using GraphPad Prism 7 Software. 

### 2.5. Cell Death Assay

At the end of 7 days of exposure to BMs and NAB-PTX in 96-well substrate-coated plates at a density of 1.5 × 10^3^ cells/well, cells were incubated overnight with ethidium homodimer (Sigma-Aldrich) at 10 mg/mL to evaluate the fluorescence of death cells (O.D. 650 nm). Images were acquired with a fluorescence microscope (Olympus). The analysis of pixel density was quantified with the ImageJ 1.46r software [68].

### 2.6. Extracellular Glucose and Lactate and cell ATP Content Assays

Cells were plated in 24-wells in 2D conditions or in ECMs-coated plates at a density of 9 × 10^5^ cells/well. For basal analysis of metabolic parameters, 2D cells were used as control. For the analysis of metabolic parameters in cells exposed to BMs, untreated cells were used as control. Cells were treated with the above BM concentrations and the cell culture medium was collected after the respective incubation time for glucose and lactate quantification. Glucose and lactate were quantified using commercial kits (Sigma-Aldrich), according to the manufacturer’s protocols. Results are expressed as ∆mM metabolite/proliferative cells. Simultaneously, after ECM removal [67], the cells were used to quantify intracellular ATP. ATP was measured using a commercial kit according to the manufacturer’s instructions (Abcam). ATP concentration in each sample was determined through the calibration curve constructed for each assay. The ATP content was expressed as total ATP normalized to the concentration of protein analyzed previously. The results presented correspond to the average of at least three independent experiments.

### 2.7. Quantification of Amino Acids in the Growth Medium by HLPC

The medium removed from the untreated, control samples or samples treated with the above concentration of BMs, as well as standards, were first deproteinized by treating them with 10 volumes of methanol. The samples were incubated on ice for 30 min and centrifuged at 12,000 rpm for 20 min at 4 °C and the supernatant was removed and stored at −20 °C until use. Prior to injection, the analytes were derivatized with OPA-MPA (ortho-phthalaldehyde, Sigma-Aldrich) and 2-mercaptoethanol (MPA) as reducing agent. Derivatization was carried out by mixing 10 μL of each sample with 48 μL of the derivatization reagent (0.2 M borate buffer, 60 mM MPA, pH 9) in a total volume of 190 μL. The separation of the derivatized analytes was obtained by HPLC (Waters Alliance Separations Module 2695) with a guard cartridge and a Kinetex 5 µm C18 150 × 4.6 mm column (Phenomenex Inc., Torrance, CA, USA). Separation was carried out at a flow rate of 1.3 mL/min using a linear gradient elution with a mobile phase consisting of 0.05 M acetate /methanol (95/5) as a polar phase (eluent A, pH 7.2) and a 0.1 M sodium acetate/methanol/acetonitrile (46/44/10) as nonpolar phase (eluent B, pH 7.2). The following gradient was applied: start 0% B, 3 min 2% B, 7 min 15% B, 17 min 50% B, 22 min 100% B (flow increased to 1.8 mL/min and hold for 5 min), 30 min 0% B (flow rate decreased to 1.3 mL/min) and hold for 5 min for equilibration. For the detection, a Waters 2475 fluorescence detector (337 nm excitation, 453 nm emission) was employed.

### 2.8. Bioenergetic Modulator (BM) Effect on NAB-PTX Cytotoxicity

A total of 1.5 × 10^3^ cells/well were seeded into 96-well in ECM-coated plates and treated with NAB-PTX (10 nM) alone or combined with the above concentrations of BMs for 7 days. Untreated cells were used as control. The effect of NAB-PTX alone and NAB-PTX + BMs on cell proliferation and cell death were evaluated as above. The results presented correspond to the average of at least three independent experiments. The combined effect of the drugs was determined using the CalcuSyn Software version 2.0 (Biosoft, Cambridge, UK). Synergy or antagonism was quantified by the combination index (CI), where CI = 1 indicates an additive effect, CI < 1 indicates synergy and CI > 1 indicates antagonism.

### 2.9. Western Blotting Analysis

Cells were seeded in 2D and on three different ECM mixes. Cells were lysed directly from 2D monolayers, while for 3D culture systems, cells were extracted from the ECM by the use of CellSperse (Cultrex) to digest Matrigel, and collagenase type I for collagen I as previously described [67,68]. After the extraction, cells were lysed in lysis buffer (HEPES 5 mM, EDTA 0.5 mM, pH 7.2 supplied with protease inhibitor 2 μL/mL, phenyl methanesulfonylfluoride (PMSF) 1 mM, sodium orthovanadate 1 mM, dithiothreitol (DTT) 1 mM, Nonidet 0.1%). Proteins were measured with Bradford (Pierce), resuspended in sodium dodecyl sulfate (SDS) sample buffer (6.25 mM Tris-HCl, pH 6.8, containing 10% (*v*/*v*) glycerol, 3 mM SDS, 1% (*v*/*v*) 2-mercaptoethanol and 0.75 mM of bromophenol blue), run on 10% SDS-PAGE, blotted to Immobilon P and analyzed by Western blotting with a polyclonal antibody against MCT1 (Millipore AB3538P), MCT4 (Santa Cruz Biotechnology, Inc., Santa Cruz, CA, USA, (H90) sc-50329) or GLUT1 (Sigma-Aldrich 07-1401) and monoclonal anti-GLUT3 (Santa Cruz Biotechnology, Inc. (G-5) sc-74399). Each blot was scanned with an Epson V600 scanner and the relative optical density of each band was analyzed using ImageJ as per [62,67,68]. Original blots can be found in Supplementary File S1.

### 2.10. Statistical Analysis

The GraphPad prism 7 software was used, with either the Student’s *t*-test or one-way ANOVA followed by the Dunnett test, considering significant values to be *p* ≤ 0.05.

## 3. Results

### 3.1. ECM Composition Is Involved in the Regulation of Metabolic Plasticity, Especially in CSCs

**Figure 1:**
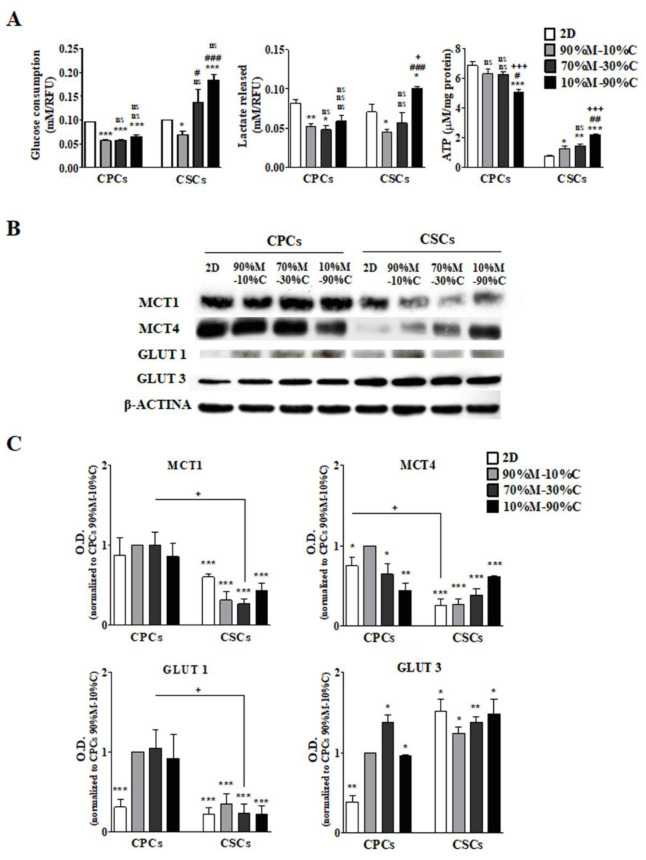
Metabolic profiles of PDAC cells in 2D and in 3D in different ECM compositions. (**A**) After 7 days of growth in the different ECMs, the growth medium was collected and basic metabolic parameters such as glucose consumption, lactate release and ATP production were measured. Results are presented as mean ± SEM in triplicate of at least three independent experiments. Significantly different between groups: * *p* < 0.05; ** *p* < 0.01; *** *p* < 0.001 compared to 2D condition of each cell line. # *p* < 0.05; ## *p* < 0.01; ### *p* < 0.001 and ns. *p* > 0.05 compared the 3D ECMs of each cell line. (**B**) Expression of the major transporters responsible for glucose consumption and lactate release. Upper panels display representative Western blots for the indicated proteins and lower panels (**C**) show a quantitative analysis of their relative expression as standardized to CPCs cells on 2D. Significantly different between groups: * *p* < 0.05; ** *p* < 0.01; *** *p* < 0.001 compared to 2D CPCs. ### *p* < 0.001 compared 2D CSCs; + *p* < 0.05; +++ *p* < 0.001 comparing CPCs to CSCs on the same ECM.

To analyze the role of the ECM composition on PDAC cells’ metabolic profile, intracellular ATP content and extracellular lactate and glucose levels were measured for both CPCs and CSCs grown in 2D or in 3D on three different ECM mixes. In Figure 1A, it can be seen that both cell lines present distinct metabolic profiles that were differently dependent on ECM composition. 

#### 3.1.1. CPCs

In 2D culture, the CPCs presented a canonical Warburg glycolytic phenotype, while in 3D growth, both glucose consumption and lactate production were reduced compared to 2D and displayed similar levels in all three ECM compositions. However, as seen in Table 1, the ratio of lactate release to glucose consumption was essentially unchanged from 2D to all 3D growth conditions, suggesting that the canonical Warburg glycolytic phenotype was maintained in all 3D ECM compositions. The cellular ATP content decreased significantly compared to 2D cultured cells only in the 10%M–90%C ECM. This, together with the lower consumption of glucose and production of lactate, suggests that these cells can utilize both OXPHOS and aerobic glycolysis in these conditions. These trends were confirmed via Western blots (Figure 1B,C), where GLUT1, GLUT3 and MCT1 expression were almost identical in all 3Ds. Interestingly, MCT4 expression in the CPCs decreased as ECM collagen I concentration increased, suggesting the possible participation of the CPCs in a reverse Warburg/lactate shuttle phenomenon (e.g., metabolic symbiosis), in which the CPCs utilize lactate produced by other cell types via MCT1 while reducing their expression of the efficient lactate exporter, MCT4 [71,72,73,74,75,76].

#### 3.1.2. CSCs

The CSCs displayed a more complex pattern and the ECM collagen I content had a greater influence on their metabolic behavior. When grown in 90%M–10%C, their rates of both glucose consumption and lactate production were greatly reduced compared to 2D. In contrast, as the percentage of ECM collagen I increased, both glucose consumption and lactate release increased stepwise such that, in 10%M–90%C, both rates were higher than in 2D. However, as seen in Table 1, the ratio of lactate release to glucose consumption decreased significantly as collagen I increased, suggesting that ECM collagen I induced a complex metabolic rewiring in the CSCs. Cellular ATP content increased immediately in 90%M–10%C compared to 2D and further increased stepwise as collagen I content increased. Western blot analysis (Figure 1B,C) showed that in CSCs, GLUT1 and GLUT3 expression were essentially identical in 2D and in all 3Ds. Interestingly, while MCT1 expression decreased from 2D to 3D and then remained essentially stable, MCT4 expression increased as ECM collagen I concentration increased. This was opposite to what occurred for MCT4 in the CPCs and suggests that the high lactate production by the CSCs in collagen I-rich ECMs could be supplying lactate to the CPCs for their metabolic use, determining a reverse Warburg effect (lactate shuttle) as hypothesized above [71,72,73,74,75,76]. 

### 3.2. High ECM Collagen I Percentage Increases Glutamine Consumption and Glutaminolysis in CSCs

The large increase in ATP production in the CSCs could indicate an increasing dependence on glutamine uptake and its use in glutamine-driven oxidative phosphorylation (glutaminolysis), as recently reported in pluripotent stem cells [77,78], leukemia CSCs [33,34] and in PDAC cells, where the primary amino acid taken up was glutamine [35,36,59,79,80], including PDAC-initiating cells [25]. However, while PDAC is characterized by high levels of glutamine uptake, the role of the increasing collagen I during the desmoplastic reaction in driving this glutamine uptake/usage has not been described.

**Figure 2:**
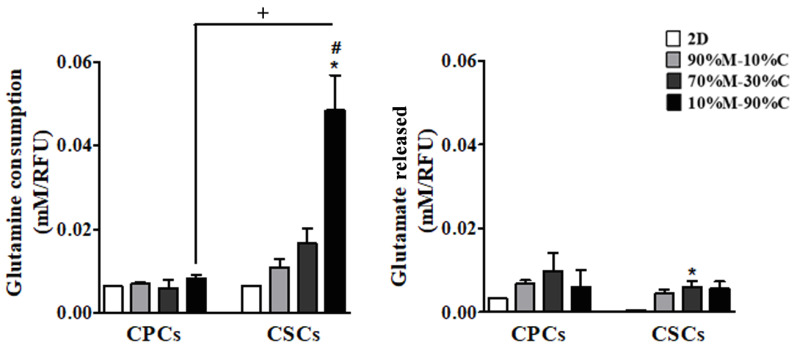
Amino acid quantification by HPLC in PDAC cell lines. The growth medium was collected after 7 days of growth in different substrates and the amino acids quantified by HPLC. Results are presented as mean ± SEM in triplicate of at least three independent experiments. Significance between groups: * *p* < 0.05; compared to 2D condition of each cells; # < 0.05 compared to 3Ds; + < 0.05 comparing CPCs to CSCs on the same ECMs.

Therefore, the medium concentration of several amino acids were analyzed in both 2D and 3D growth conditions using HPLC analysis and, by far, the largest fluxes were observed for the non-essential amino acids glutamine and glutamate (Figure 2). Glutamine consumption was similar for both CPCs and CSCs in 2D; and while collagen I content did not influence glutamine consumption in the CPCs, in the CSCs, glutamine consumption increased stepwise with increasing ECM collagen I content. Further, while glutamate release increased in 3D compared to 2D for both cell types, the ECM collagen I content had no influence on glutamate release in either cell type. 

An analysis of the ratio of both glutamate release to glutamine uptake (Table 2) and of lactate release to glutamine uptake (Table 3), revealed that in 3D, as ECM collagen I content increased, these ratios remained stable in the CPCs while decreasing in the CSCs. Indeed, in these conditions, the ratio of glutamate release to glutamine uptake was greatly reduced only in the CSCs, suggesting a major shift in these cells in the utilization of glutamine. This also demonstrates that it is the CSCs that display metabolic amino acid plasticity, as only they shifted their metabolic dependences toward anaplerotic OXPHOS glutamine metabolism (glutaminolysis) as the collagen I content of the ECM increased.

Altogether, these data suggest that while the CPCs remained essentially reliant on glycolysis in all growth conditions, in the CSCs, ECM collagen I induced a more complex metabolic pattern in which they were able to switch their metabolic dependence from glucose toward glutamine. This is different from leukemia stem cells, which are completely dependent on metabolizing amino acids for energy [59,78]. 

### 3.3. Treatment with Bioenergetic Modulators (BMs) Affects Cell Growth and Survival of PDAC Cells

The growth and levels of death of the two PDAC cell lines (CPCs and CSCs) were evaluated after metabolic remodeling through the treatment with three different bioenergetic modulators (BMs): the glycolysis inhibitor 2-DG (5 mM), the PDH kinase inhibitor DCA (20 mM) and the mitochondrial complex I inhibitor, phenformin (0.01 mM). In 2D, CPC growth was inhibited primarily by the glycolytic inhibitors (54 ± 6%, 37 ± 5% and 9 ± 4% inhibition for 2DG, DCA and phenformin, respectively), while the opposite was true for the CSCs (4 ± 3%, 2 ± 5% and 41 ± 2% inhibition for 2DG, DCA and phenformin, respectively). These data are shown in a graphical format in Supplemental Figure S1 and support the basic pattern observed for the two cell lines reported in Figure 1.

**Figure 3:**
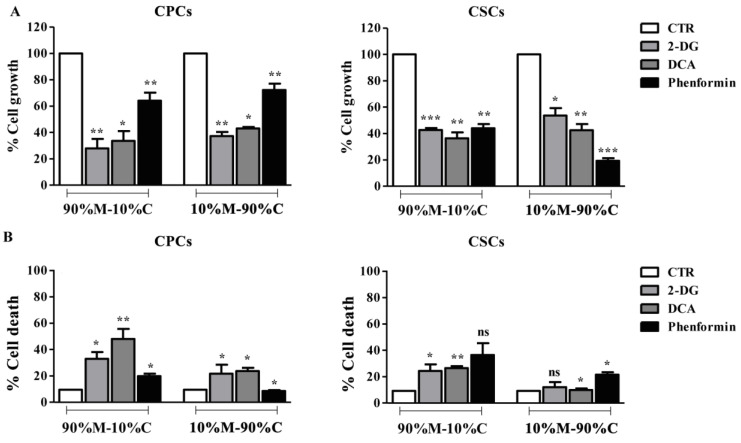
Effect of bioenergetic modulators (BMs), 2-DG, DCA and phenformin on PDAC cell proliferation and cell death. After 7 days of treatment with BMs (2-DG: 5 mM, DCA: 20 mM and phenformin: 0.01 mM) in different substrates, (**A**) cell growth was measured by the resazurin assay. (**B**) Cell death was quantified using the ethidium homodimer assay where the integrity density of dead or dying cells (stained red) was measured by the ImageJ software. Untreated cells were used as control. Results are presented as the mean ± SEM of triplicates from at least three independent experiments. Significance between groups: * *p* < 0.05; ** *p* < 0.01; *** *p* < 0.001 compared to untreated cells; ns: not significant.

In 3D, we measured the effect of the BM compounds in the two extreme ECM compositions (90%M–10%C vs. 10%M–90%C). As can be seen in Figure 3, all the BM compounds reduced cell proliferation in both cell lines, demonstrating that 3D growth increases their plasticity for dependence on both glycolytic and OXPHOS pathways. However, in the CPCs (Figure 3A, left panels), the glycolytic agents still induced a much higher inhibition of cell growth compared to phenformin, and these effects were independent of ECM composition. In the CSCs (Figure 3A, right panels), ECM collagen I content differentially modified the effect triggered by the BMs, in that growth was similarly inhibited by all three compounds in 90%M–10%C, while in 10%M–90%C, the inhibitory effect of phenformin was 2-fold higher than with the anti-glycolytic compounds.

Cell death in the CPCs (Figure 3B, left panel) was much more strongly induced by the glycolytic inhibitors than phenformin on both ECMs, while in the CSCs (Figure 3B, right panel), phenformin had a stronger cytotoxic effect, especially in 90%M–10%C, where neither glycolytic inhibitor had any effect, but also in 10%M–90%C, where the effects of the glycolytic inhibitors were minimal. 

Therefore, we hypothesize that both lactic fermentation (glycolysis) and OXPHOS are important for CSC proliferation and mortality when growing in 90%M–10%C, while OXPHOS dominates these phenotypes in 10%M–90%C. The dependence of CSCs on respiratory metabolism (OXPHOS) is supported by the enhanced glutamine consumption observed in Figure 2 and Table 2 and Table 3. 

### 3.4. The Disruption of Energetic Pathways Synergistically Potentiates NAB-PTX Action

We lastly investigated the influence of these BMs on the efficacy of the currently utilized conventional PDAC antitumor drug, NAB-Paclitaxel (NAB-PTX), with the objective to increase its efficacy and overcome treatment resistance. To evaluate the combined effect of NAB-PTX with BMs, both CPCs and CSCs were treated with 10 nM NAB-PTX alone or simultaneously with each BM for 7 days with untreated cells as control.

**Figure 4:**
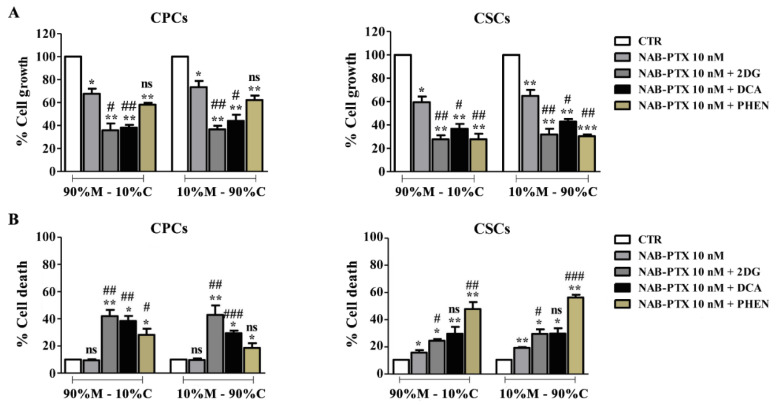
Effect of BM treatment significantly synergizes with NAB-PTX cytotoxicity in PDAC cell growth (**A**) and death (**B**) in different ECMs. Cells were exposed with a fixed concentration of BMs (2-DG (5 mM), DCA (20 mM) and phenformin (Phen. 0.01 mM)) and NAB-PTX (10 mM), during the respective incubation time. Results represent the mean ± SEM of triplicates from at least three independent experiments. Significance between groups: * *p* < 0.05; ** *p* < 0.01; *** *p* < 0.001 compared to untreated cells. # *p* < 0.05; ## *p* < 0.01; ### *p* < 0.001 compared to cells treated only with NAB-PTX 10 nM; ns: not significant.

As seen in Figure 4A, NAB-PTX treatment alone inhibited cell growth similarly in both cell lines and on both ECMs. The combined treatment of NAB-PTX with the BMs effectively enhanced the inhibition of cell growth by NAB-PTX for most cases. Again, in the CPCs, the glycolytic inhibitors more strongly potentiated the inhibitory effect of NAB-PTX on growth than phenformin, while in the CSCs, all three BMs potentiated the inhibitory effect of NAB-PTX about equally in both ECMs, although DCA had a somewhat lower effect than the other BMs.

Regarding cell death (Figure 4B), treatment with NAB-PTX alone did not induce a significant cytotoxic effect in the CPCs while producing a low but significant increase in death on both ECMs in the CSCs. In the CPCs, all the BMs, but especially the glycolytic inhibitors, significantly increased NAB-PTX-induced cell death and, again, this effect was independent of the ECM composition. In CSCs, the OXPHOS inhibitor phenformin, combined with NAB-PTX induced the highest rate of cytotoxicity with a slightly higher effect in 10%M–90%C. These results support the previous data (Figure 1 and Figure 3) that the CPCs are more dependent on the glycolytic pathway, since the glycolytic inhibitors induced higher levels of death in these cells when combined with NAB-PTX. In contrast, the CSCs were more sensitive to the OXPHOS inhibitor, phenformin, supporting the hypothesis that they have a larger dependence on OXPHOS and mitochondrial pathways.

The CalcuSyn program (Biosoft, Cambridge, UK) was used to analyze if these drugs had an additive or synergistic effect in combination with NAB-PTX by calculating the combination index. As can be seen in Table 4, all BMs produced a synergistic effect on both growth and cytotoxicity in both cell lines when in combination with NAB-PTX. However, in the CSCs, the treatment with the highest level of synergism was phenformin plus NAB-PTX. Therefore, the combination of BM compounds with NAB-PTX can overcome the relative resistance to standard NAB-PTX treatment in more aggressive conditions, such as the presence of desmoplastic environment and in the CSC population.

## 4. Discussion

The ECM plays many functions, including acting as a physical scaffold, facilitating interactions between the different cell types, providing survival and differentiation signals and resistance to anticancer drugs [81]. Further, the ECM composition is now known to be able to override cancer cell intrinsic signaling to regulate tumor progression independently of the clonal heterogeneity of the tumor and is one of the major drivers of heterogeneity, metastatic capacity, plasticity and therapy resistance risk/prognosis in cancer cells [82,83], including PDAC [67,68,84,85]. Indeed, the desmoplastic reaction, characterized by an intense deposition of collagen and one of the main features of PDAC, is related to a more invasive and aggressive phenotype and with resistance to therapy [6,8,86]. The low survival rates and the dismal results presented by the available conventional treatments in PDAC can also be related to the interaction of this desmoplastic extracellular environment with the CSC subset of tumor cells [87]. Understanding how the ECM component of the tumor microenvironment influences cancer cell behavior will lead to the better comprehension of PDAC biology and to the identification of new targets that may improve the treatment of pancreatic cancer patients. CSCs represent a small group of cells that are considered to be the main cause of metastasis and treatment resistance, due to their DNA repair systems, relative quiescent behavior, and ability to form new tumors. Therefore, the targeted eradication of these cells will be an important advance in PDAC therapy. 

The altered metabolism that distinguishes cancer cells from their normal counterparts is a novel paradigm in cancer treatment, and new and effective compounds targeting the metabolic alterations in cancer cells have been developed and approved in clinical trials [88,89]. Like the parenchymal cancer cell population (CPCs), CSCs also have a reprogrammed metabolism [90]; however, much less is known concerning CSC metabolism and its regulation. The role of the interaction of the cancer cell with the extracellular matrix in determining their metabolic phenotype and the cross-talk between the two are still very poorly understood [91].

Data concerning CSC metabolism is controversial with some reports that also CSCs use the same energetic pathway(s) as the non-stem CPCs, e.g., lactic fermentation [32], while others have demonstrated that OXPHOS is the main source of energy for CSCs [28,43]. It has been demonstrated that CSCs show a high metabolic plasticity and can readily adapt their metabolism according to the microenvironment conditions, tissue of origin and state of differentiation [32,91]. Indeed, CSCs are mainly oxidative in the quiescent state, and in the proliferative state, CSCs have a higher capacity to change their metabolic necessities, such that they have a higher metastatic ability, resistance to therapy and a combined phenotype that relies upon glycolysis and OXPHOS simultaneously [28,43]. 

Therefore, the main objective of the present study was to understand how ECM composition influences the metabolic behavior of PDAC parenchymal cells and CSCs. To this end, we measured glucose and amino acid consumption, lactate secretion and the expression of their principal transporters together with ATP production in 2D growth and 3D growth in different ECM substrates. This was performed in both control conditions and in the presence of the following bioenergetic modulators (BMs) that target different metabolic pathways: (i) the anti-glycolytic agent 2-deoxyglucose (2-DG, inhibitor of the HK); (ii) dichloroacetate (DCA), an inhibitor of PDH kinase that activates pyruvate oxidation decreasing the Warburg effect; (iii) and an inhibitor of the OXPHOS mitochondrial complex I, phenformin. Lastly, to study the influence of ECM and the role of metabolism in the response to therapy, we determined the effect of the combination of each of these BMs with nab-paclitaxel (NAB-PTX) on the two ECMs.

We observed that the desmoplastic microenvironment can be decisive in the selection of the metabolic course: increasing levels of collagen I in the ECM drove a higher metabolic plasticity in the CSCs, while the CPC metabolic phenotype was more independent of the ECM composition, although some subtle differences were also observed. When grown in 2D, the CPCs exhibited Warburg metabolism with consumption of glucose and production of lactate and ATP and, indeed, in 2D, the effect of the BMs on growth and cell death supported this conclusion. However, in 3D and especially in the 90%M–10%C, a distinct pattern was found in which both the lactic fermentation and OXPHOS pathways can be used. Indeed, as seen in Figure 3, in CPCs both anti-glycolytic agents, 2-DG and DCA, strongly decreased proliferation by inducing cell death independently of ECM composition, while phenformin determined a much lower inhibition of cell growth and stimulation of cell death, although these effects were higher than in 2D. Therefore, we conclude that, in 3D culture, while glycolysis is the main energetic pathway used by the CPCs, the mitochondria also seem to play a minor role as indicated by the effect of the OXPHOS inhibitor, phenformin. 

On the contrary, the CSCs presented a very distinct metabolic pattern that was strongly influenced by the ECM composition. As reported in the literature, the CSCs displayed a strong ability to modify their metabolic characteristics according to the environment conditions [51,52,53,54]. As the ECM collagen I increased, the CSCs had a higher consumption of glucose, release of lactate and ATP production, but with a decreasing ratio of lactate release to glucose consumption, suggesting a rewired metabolism. Indeed, on collagen I-enriched ECM, the CSCs consumed ever more glutamine with, however, strong reductions in the ratio of both glutamate and lactate released to glutamine consumption. Together with the stepwise increase in ATP, these data strongly point to an increase in glutaminolysis as collagen I increases in the ECM with a shift in its metabolic pathway end products. In this respect, while all the BMs were able to reduce CSC proliferation and increase cell death, phenformin induced the highest inhibition of proliferation and increase in cell death, especially in the ECM composition that had the highest level of glutamine uptake. 

The inhibition of metabolic dependence by using metabolic inhibitors has been tested in different types of cancers and their derived CSC populations. For example, breast cancer CSCs were shown to be very sensitive to 2-DG and, furthermore, 2-DG showed a synergistic effect with the commonly used doxorubicin in breast cancer therapy, which further reduced the stemness of the cell population [92]. However, other studies have shown the importance of the mitochondrial complex respiration in the CSCs of many types of cancers and a high dependence on this mitochondrial activity even in cells with mutations that impaired the TCA cycle. Indeed, OXPHOS plays an important role in the maintenance of CSC stemness [43,93,94] and the anti-diabetic drug, and the OXPHOS inhibitor, metformin, leads to a reduction in the stem cell pool and in in vivo tumor growth in pancreatic/breast [54] and prostate CSCs [95]. Indeed, mitochondria are still important organelles for the use of other precursors, for example, glutamine via glutaminolysis [96]. Other recent reports have shown the importance of metabolic sources other than glucose to maintain the tumorigenicity of CSCs, as they are able to use the amino acids glutamine and alanine to support their mitochondrial energy production [33,34,97]. For this reason, the use of compounds that block these metabolic pathways can be of great importance in antitumor therapy.

Indeed, our results show that in PDAC, it is the CSC population that has a higher consumption of glutamine, and that this increase in consumption is driven by collagen I levels in the ECM. In this respect, Li and co-authors showed that decreasing the glutamine concentration present in the microenvironment through the inhibition of enzymes responsible for glutamine metabolism reduces stemness and sensitizes the CSCs to radiotherapy in vitro and in vivo [96]. We verified in this study, that CSC grown in 10%M–90%C have an increased glucose consumption, production of lactate and ATP, but also a very large increase in glutamine consumption, indicating a sustained mitochondrial activity. 

Consequently, targeting not only glycolysis, but also mitochondrial functionality will expand the search for novel anticancer drugs against tumorigenesis and chemoresistance, and a combination of mitochondria-targeted agents with conventional chemotherapeutic drugs may be required to achieve the maximum efficacy to disrupt the CSC population and to improve the current therapies [45,46].

In this respect, our results clearly showed that the combination of the conventional drug NAB-PTX with BMs increased the effect of the standard therapy in all the conditions. It is important to note that in both the CPC and CSC cells, the anti-glycolytic compounds together with NAB-PTX induced a stronger effect on cell death compared with NAB-PTX treatment alone, independently of ECM composition, and this effect is synergistic (Table 4). Importantly, in the CSCs, the treatment with the highest level of synergism was phenformin plus NAB-PTX. Altogether, these data further support our hypothesis that ECM composition directly influences basic metabolism differently in the CPCs and CSCs with collagen I in the ECM driving a higher metabolic plasticity in the CSCs. Further, we find that the BMs both alone and especially in combination with current treatment procedures can be an emergent strategy in PDAC treatment and in the abolishment of the CSC population.

## 5. Conclusions

We conclude that ECM composition directly influences basic metabolism differently in the CPCs and CSCs with collagen I in the ECM, driving a higher metabolic plasticity in the CSCs. Interestingly, a possible hypothesis can be that the high lactate production by the CSCs in collagen I-rich ECMs could be supplying lactate to the CPCs for their metabolic use, which is supported by the levels of MCT1 and MCT4 present in CSCs and CPCs in collagen I-rich ECMs. Indeed, MCT1 is a monocarboxylate transporter that mediates the uptake of lactate into the cell, whereas MCT4, due to its high Km, is mainly involved in lactate efflux. In this way, as CSCs presented higher levels of MCT4, this indicates that they export higher levels of lactate that can be used by CPCs as substrate, as these cells present higher levels of MCT1, involved in lactate uptake.

Further, we find that the BMs both alone and especially in combination with current treatment procedures can be an emergent strategy in PDAC treatment and in the abolishment of the CSC population. Indeed, the results clearly showed that the combination of the conventional drug NAB-PTX with BMs increased the effect of the standard therapy in all conditions. Indeed, in both the CPC and CSC cells, the anti-glycolytic compounds together with NAB-PTX induced a stronger effect on cell death compared with NAB-PTX treatment alone, independent of ECM composition, and this effect is synergistic (Table 4). Importantly, in the CSCs, phenformin plus NAB-PTX was the treatment with the highest level of synergism. Further, we find that the BMs both alone and especially in combination with current treatment procedures can be an emergent strategy in PDAC treatment and in the abolishment of the CSC population. 

Consequently, targeting not only glycolysis, but also mitochondrial functionality will expand the search for novel anticancer drugs against tumorigenesis and chemoresistance, and a combination of mitochondria-targeted agents with conventional chemotherapeutic drugs may be required to achieve the maximum efficacy to disrupt the CSC population and to improve the current therapies.

## Figures and Tables

**Table 1:** Ratio of lactate released to glucose taken up.

	CPCs	CSCs
**2D**	0.86 ± 0.04	0.72 ± 0.04
**90%M–10%C**	0.90 ± 0.03	0.70 ± 0.07
**70%M–30%C**	0.88 ± 0.05	0.64 ± 0.02 *
**10%M–90%C**	0.92 ± 0.06	0.56 ± 0.03 **

The ratio of mM of lactate released to mM of glucose consumed. CPCs: Cancer parenchymal cells; CSCs: Cancer stem cells. * *p* < 0.05, ** *p* < 0.01 compared to 2D counterparts.

**Table 2:** Ratio of glutamate released to glutamine taken up.

	CPCs	CSCs
**2D**	1.24 ± 0.72	0.53 ± 0.14
**90%M–10%C**	0.94 ± 0.1	0.41 ± 0.01
**70%M–30%C**	1.01 ± 1.32	0.37 ± 0.01 *
**10%M–90%C**	0.76 ± 0.47	0.12 ± 0.01 **

The ratio of mM of glutamate released to mM of glutamine consumed. * *p* < 0.05, ** *p* < 0.01 compared to compared to 2D growth.

**Table 3:** Ratio of lactate released to glutamine taken up.

	CPCs	CSCs
**2D**	12.64 ± 0.70	10.59 ± 1.44
**90%M–10%C**	7.39 ± 0.38	4.13 ± 0.35
**70%M–30%C**	7.94 ± 0.94	3.84 ± 0.49
**10%M–90%C**	7.08 ± 0.92	2.04 ± 0.15

The ratio of mM of lactate released to mM of glutamine consumed.

**Table 4:** Combination index (CI) values determined from treatment with NAB-PTX and BMs.

		90%M–10%C	10%M–90%C
**CPCs**	**2-DG**	<1 (0.58)	<1 (0.66)
	**DCA**	<1 (0.65)	<1 (0.52)
	**Phenformin**	<1 (0.79)	<1 (0.86)
**CSCs**	**2-DG**	<1 (0.48)	<1 (0.57)
	**DCA**	<1 (0.59)	<1 (0.66)
	**Phenformin**	<1 (0.32)	<1 (0.27)

Combination index (CI) values were determined from cell treatment with NAB-PTX (10 nM) and BMs (2-DG: 5 mM; DCA: 20 mM and phenformin: 0.01 mM) in monotherapy compared with the combination. CI = 1 indicates an additive effect, CI < 1 indicates synergy, and CI > 1 indicates antagonism.

## Data Availability

Data available on request due to restrictions.

## Supplementary Materials

The following supporting information can be downloaded at: https://www.mdpi.com/article/10.3390/cancers15153868/s1, Figure S1: Representative Western blot of Gluts and MCTs in CPCs and CSCs growing as 2D or 3D organotypic cultures on different ECMs; File S1: Original blots.
