# Supplementary figures and images for: Extracellular Matrix Collagen I Differentially Regulates the Metabolic Plasticity of Pancreatic Ductal Adenocarcinoma Parenchymal Cell and Cancer Stem Cell
Source: Cancers (Basel). 2023 Jul 29;15(15):3868. doi: 10.3390/cancers15153868 (PMC10417137; doi:10.3390/cancers15153868)

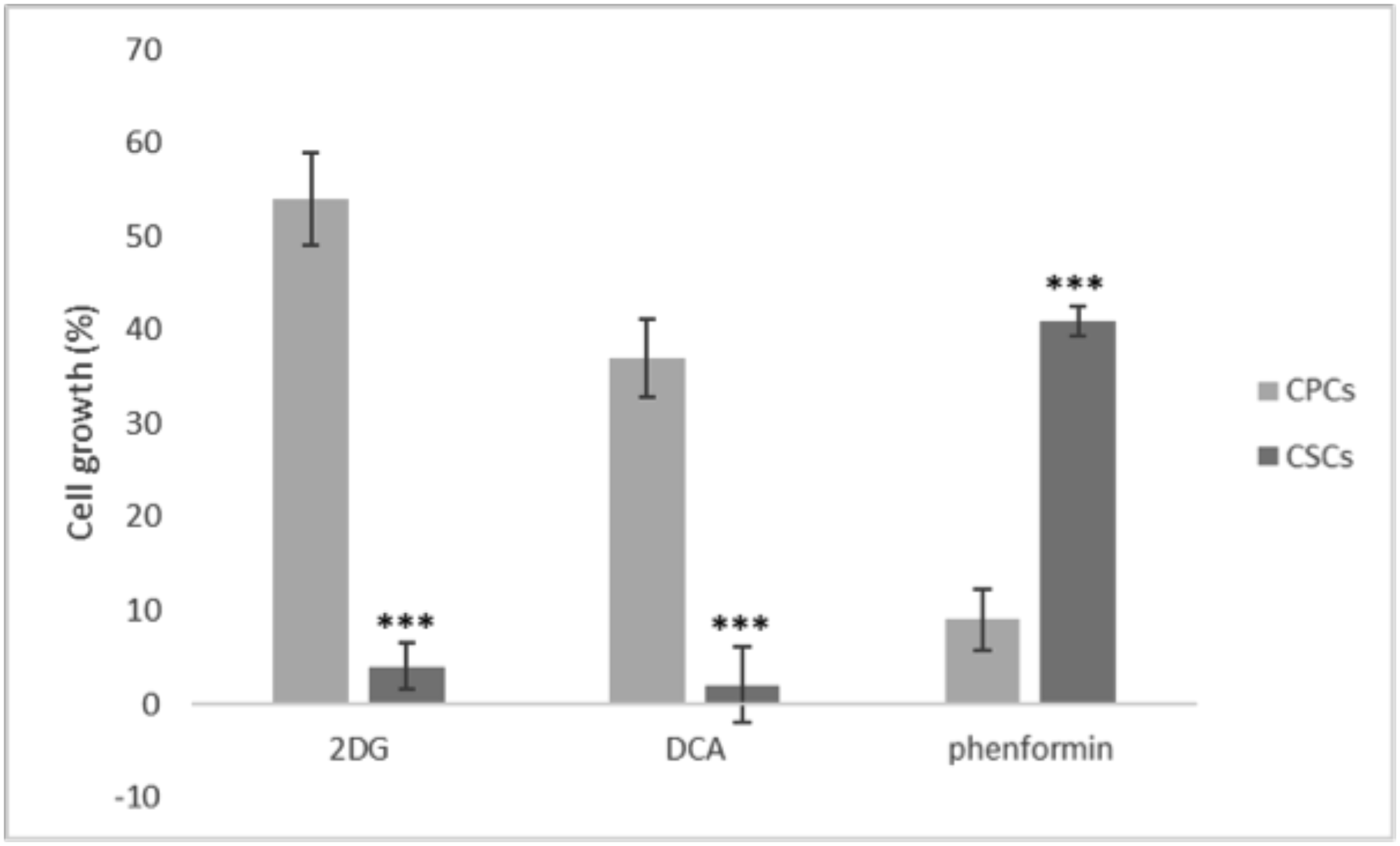

Supplement: Supplementary file 1 [file cancers-15-03868-s001.zip › cancers-2440991-supplementary/cancers-2440991-Figure S1.tif]

# Gluts

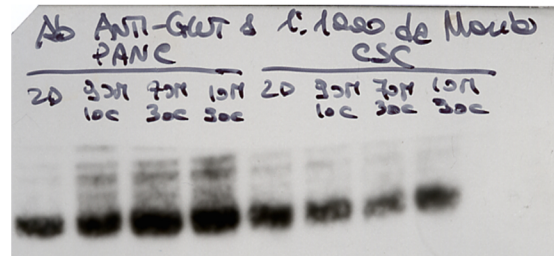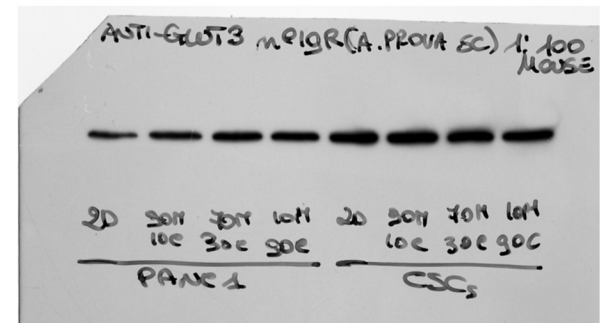

# MCTs

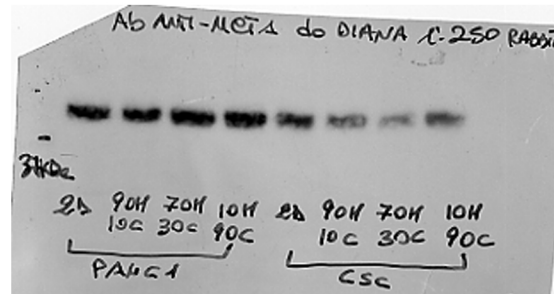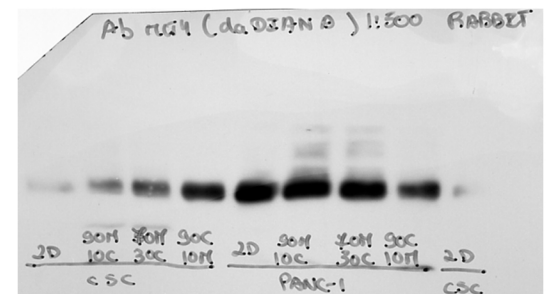

Note that sample loading of MCT4 is opposite that of the other gels

# Actin

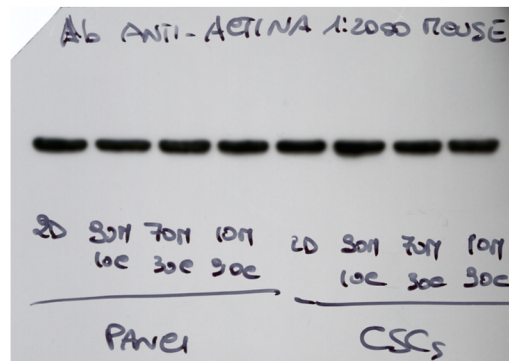

Supplement: Supplementary file 1 [file cancers-15-03868-s001.zip › cancers-2440991-supplementary/cancers-2440991-File S1 - original-images.pdf]
